# Daptomycin and Ceftaroline Combination Therapy in Complicated Endovascular Infections Caused by Methicillin-Resistant Staphylococcus epidermidis

**DOI:** 10.7759/cureus.54134

**Published:** 2024-02-13

**Authors:** Sílvia Policarpo, Raquel Duro, Nuno R Pereira, Lurdes Santos

**Affiliations:** 1 Department of Infectious Diseases, Centro Hospitalar Universitário de São João, Porto, PRT; 2 Department of Infectious Diseases, Centro Hospitalar do Tâmega e Sousa, Penafiel, PRT; 3 Department of Medicine, Faculdade de Medicina da Universidade do Porto, Porto, PRT

**Keywords:** ceftaroline, daptomycin, antimicrobial combination therapy, methycillin-resistent staphylococcus epidermidis, endovascular infections

## Abstract

Background

In complicated endovascular infections by methicillin-resistant *Staphylococcus aureus *(MRSA) or *Staphylococcus epidermidis *(MRSE), when first-line therapy with vancomycin (VAN) or daptomycin (DAP) fails, combination therapy with ceftaroline (CFT) and DAP has been shown to be a useful approach as salvage therapy for persistent MRSA bacteremia.

Objectives

This study aimed to describe experience with daptomycin and ceftaroline combination therapy in MRSE-complicated endovascular infections.

Methods

A single-center retrospective review of consecutive patients with MRSE-complicated endovascular infections treated with ≥72 hours of DAP+CFT at any time during the course of treatment, from January 1, 2016 to December 31, 2020, at Centro Hospitalar Universitário São João (CHUSJ), Porto, Portugal, was conducted. The exclusion criteria were known resistance to daptomycin or ceftaroline, total time of combination therapy <72 hours and loss to follow-up.

Results

We identified seven cases that matched our criteria: five endocarditis and two central venous catheter infections. Six patients switched to combination therapy due to treatment failure with first-line agents - three due to persistent bacteremia and three due to progression of infection despite negative blood cultures. Effective surgical source control took one to four weeks to occur. Three patients died during the treatment, one from progression of the disease and two due to another infection.

Conclusions

We consider the DAP+CFT combination therapy to be a valid and safe therapeutic choice in complicated patients, such as those with severe infection, poor functional status, and impossibility or delay of surgical source control. However, conclusions on the role of combination therapy should be careful due to the low number of patients and the several confounding factors.

## Introduction

Endovascular infections are mainly caused by gram-positive cocci, namely, *Staphylococcus aureus* (*S. aureus*) and coagulase-negative staphylococci (CoNS), such as *Staphylococcus epidermidis* (*S. epidermidis*). As the use of endovascular devices increases, complicated endovascular infections have become more frequent. The treatment of these infections can be particularly challenging, given biofilm formation, difficulties in quickly achieving source control, and the emergence of drug-resistant organisms. Evidence on the best treatment strategy for these infections by gram-positive cocci is lacking, and the one that exists is largely focused on *S. aureus*.

Over the past years, combination therapy with vancomycin (VAN) or daptomycin (DAP) plus ceftaroline (CFT) has been studied as a possible strategy for MRSA bloodstream infection (BSI). Studies have varied significantly in their design, with some authors comparing early combination treatment with standard of care (SOC) and others reporting combination treatment as an option for salvage treatment [1-4]. This available body of evidence does not allow to establish the benefit of combination therapy in all MRSA BSIs, but it does show a tendency toward the reduction of mortality and recurrence of infection in patients with complicated BSI, which might be higher in those with complicated primary endovascular infection who switch to combination therapy early on.

As hard-to-treat endovascular infections by CoNS become progressively more common, it becomes relevant to study possible strategies for their management. There are no available data on the use of VAN or DAP plus CFT combination therapy in the treatment of MRSE-complicated BSIs. In our center, we have used this approach, mostly as salvage therapy in patients with persistent infection despite adequate first-line antimicrobial therapy. Given the growing incidence of these infections, which carry important morbidity and mortality, we hereby aim to describe and reflect on our experience with this strategy.

## Materials and methods

Study design

A single-center retrospective review of consecutive patients with MRSE-complicated endovascular infection treated with daptomycin and ceftaroline combination therapy, between January 1, 2016 and December 31, 2020, at Centro Hospitalar Universitário São João (CHUSJ), Porto, Portugal, was conducted. The study protocol was approved by the CHUSJ Ethics Committee. Patients were eligible for inclusion if they met all the following criteria: i) age ≥18 years, ii) at least two positive blood cultures with MRSE growth, iii) complicated endovascular infection, and iv) treatment with ≥72 hours of DAP+CFT at any time during the course of treatment. Exclusion criteria were known resistance to either daptomycin or ceftaroline and loss to follow-up.

Definitions

"Complicated endovascular infection" is one of the following: endocarditis, presence of metastatic infection, failure to defervesce within 72 hours of appropriate therapy, persistence of positive blood cultures ≥72 hours after initiation of antimicrobial therapy, and presence of an implanted endovascular device. "Treatment failure" is the persistent bacteremia or progression of infection despite adequate antimicrobial treatment, with or without source control. "Persistent bacteremia" is the persistence of positive blood cultures >72 hours after initiation of adequate antimicrobial therapy. "Progression of infection" is the appearance of new vegetation or perivalvular abscess on echocardiography or cardiac imaging or enlargement of the ones previously known.

Statistical analysis

Categorical variables were described by frequency and continuous variables were analyzed with median value. Calculations and statistical analysis were performed on Microsoft Excel 2016 software (Microsoft Corporation, USA).

## Results

We identified nine patients who matched our inclusion criteria, of which two were excluded (one was lost to follow-up and the other presented resistance to daptomycin). 

Table 1 summarizes the details of the individual cases. Of the seven patients who were included in this analysis, four were female. Five patients were 65 years old or more, and the Charlston Comorbidity Index was ≥5 in four cases. Five patients had endocarditis and two had central venous catheter (CVC) infections, one of which was complicated by septic thrombophlebitis of the brachiocephalic vein.

**Table 1:** Details of individual cases AC: arterial catheter; AKI: acute kidney injury; CCI: Charlson Comorbidity Index; CVC: central venous catheter; CT: daptomycin and ceftaroline combination therapy; DAP: daptomycin; *E. coli*: *Escherichia coli*; HR: hypersensitivity reaction; MIC: minimum inhibitory concentration; RIF: rifampicin; PV: prosthetic valve; VAN: vancomycin *The first control blood cultures were drawn seven days after the switch to DAP + CFT and they were sterile.

Case	Age, gender, CCI	Source of infection	Devices or other heterologous material	Anti-MRSE antibiotic previous to switch to CT	Reason for switching to CT	Source control at the time of switch to CT	Days of CT	Days of bacteremia during CT	Adverse effects	Alive at 12 months? (cause of death)
1	M 83; 6	Endocarditis, spondylodiscitis, splenic embolization	Aortic PV	VAN + GEN + RIF	Progression of endocarditis	No	34	Cleared prior	No	No (different infection, *K. pneumoniae* pneumonitis)
2	M 70; 5	Endocarditis, CNS embolization	Aortic PV; mitral PV; CVC and AC (removed)	VAN, DAP	Persistent bacteremia	No	28	Cleared prior	HR (maculopapular rash and eosinophilia)	Yes
3	F 77; 6	Endocarditis	Aortic PV	VAN + GEN, DAP + GEN	Other	No	35	Cleared prior	HR (maculopapular rash)	No (mediastinitis and bacteremia by ESBL-positive *K. pneumoniae*)
4	M 52; 1	CVC; septic venous thrombosis	CVC (removed)	VAN, DAP	Persistent bacteremia	No (CVC removed but septic thrombosis was not approached surgically)	25	7*	Leucopenia with severe neutropenia	Yes
5	F 70; 6	Endocarditis	Aortic PV; mitral PV	VAN + GEN + RIF	Progression of endocarditis	No (relapse of endocarditis after valve replacement surgery)	15	Cleared prior	No	No (progression of the current infection)
6	F 68; 6	Endocarditis	Aortic PV; pacemaker	DAP + RIF	Progression of endocarditis	Yes	39	Cleared prior	No	Yes
7	M 28; 0	CVC	CVC (removed)	VAN	Persistent bacteremia	No	10	Cleared prior	No	Yes

Six patients switched to combination therapy due to treatment failure with first-line agents. Three had progression of endocarditis despite having already achieved blood culture sterilization; three had persistent bacteremia when a decision was made to switch to combination therapy (although in two of these, the blood cultures collected at the time of antibiotic switch were later known to be negative). In the other patient, who was previously under vancomycin + gentamycin, the physician’s choice to switch to combination therapy was based on the development of acute kidney failure and high vancomycin minimum inhibitory concentration (MIC of 2 mg/L).

Most patients had not achieved source control by the time of switch to combination therapy, and the time from bacteremia onset to source control ranged from one to four weeks.

Among the patients with endocarditis (patients 1, 2, 3, 5, and 6), two patients (patients 1 and 2) were deemed unsuitable for surgical valve replacement by the cardiothoracic surgery team due to high surgical risk. Patient 1 died of nosocomial pneumonia by *Klebsiella pneumoniae* before completing treatment for endocarditis, and patient 2 completed six weeks of antimicrobial therapy and showed complete resolution of the infection. Patient 3 first underwent isolated antimicrobial therapy and was switched to combination therapy in the setting of AKI (see above), but later showed progression of infection and was submitted to surgical valve replacement four weeks after bacteremia onset; she died of postoperative mediastinitis and bacteremia by ESBL-positive *Klebsiella pneumoniae* before completing treatment for endocarditis. Patient 5 underwent aortic and mitral valve replacement one week after diagnosis of endocarditis, but one month later, she showed relapsed endocarditis (without bacteremia). She was considered unfit for cardiothoracic surgery at that point, and antimicrobial therapy was optimized to combination therapy with DAP + CFT, without success; she died of progression of the infection. Finally, patient 6 initially showed a favorable evolution under antimicrobial therapy with DAP + rifampicin but control echocardiography at the third week of treatment showed progression of endocarditis; she was submitted to valve replacement and antimicrobial therapy was changed to DAP+CFT combination therapy given previous failure with a DAP-containing regimen. She completed six weeks of antimicrobial therapy after surgery, with complete resolution of the infection.

Patient 4 developed CVC-associated BSI and had persistently positive blood cultures despite the removal of the catheter and antimicrobial therapy with daptomycin. Echocardiography showed no vegetations; a previously diagnosed non-occlusive thrombus of the brachiocephalic vein was deemed to be the probable focus of persistent infections. Antimicrobial therapy was switched to DAP + CFT and he completed four weeks of antimicrobial therapy post sterilization of blood cultures, with complete resolution of the infection. Patient 7, a patient with severe Crohn’s disease under parenteral nutrition, also had CVC-associated BSI; in this case, an attempt was made to salvage the catheter. He started systemic and lock therapy with vancomycin. However, he showed persistence of positive blood cultures, and for this reason, antimicrobial therapy was switched to DAP + CFT combination therapy before the CVC was removed two days later. The last blood cultures drawn before the switch of antimicrobials (before removal of CVC) were later known to be negative.

The total time of DAP + CFT ranged from 10 to 35 (median 28 days). Three patients developed adverse effects likely related to DAP + CFT. Two patients (patients 2 and 3) had hypersensitivity reactions, which presented with maculopapular rash (and eosinophilia in one of them). Even though these reactions were not severe, given that they had been on combination therapy for at least four weeks when they occurred, CFT was suspended after a discussion with Immunoalergology. Patient 2 remained on DAP monotherapy and patient 3 continued with DAP + trimethoprim/sulfamethoxazole. Patient 3 developed leucopenia with severe neutropenia (neutrophil counts <100 cells/µL). DAP+CFT was immediately stopped, and leucopenia resolved in less than seven days, without the need for granulocyte colony-stimulating factor and without any associated infection. As he was close to completing the treatment, no other antimicrobial regimen was started.

## Discussion

*S. epidermidis* is a commensal organism found on human skin and mucous membranes. Although its isolation in blood cultures frequently represents contamination, it has also been implicated in true infections. Therefore, the interpretation of its isolation in blood cultures is not always straightforward, even in clinical scenarios where it is a plausible agent. *S. epidermidis* usually behaves as an indolent bacterium, but it can form biofilms, enabling it to persistently colonize surfaces, such as percutaneous or implanted endovascular devices [5]. 

Biofilms are complex structures involving the formation of extracellular matrix and a complex microbial organization, which result in host immune system evasion and lower response to antimicrobial therapy [6]. The difficulty of treating them solely with antimicrobial therapy is the main reason why source control with device removal is paramount in the treatment of these infections. However, in clinical practice, a longer time than recommended to source control can be explained by several factors, such as delay in diagnosis, unwillingness from the medical team to promptly remove devices (i.e., CVC in patients with poor vascular accesses), lack of availability from surgical teams to perform source control surgeries at an early timing, or even, in patients with poorer functional status and higher surgical risk, protraction of surgical approach in the hope that antimicrobial therapy alone might resolve the infection.

Methicillin resistance is found in around 80-90% of CoNS isolates [7-10], far higher than commonly seen in *S. aureus,* and seems to be increasing [11]. As in *S. aureus*, methicillin resistance in CoNS is mediated by the mecA gene, which encodes a low-affinity penicillin-binding protein (PBP 2a) [12]. Methicillin resistance is a predictor of mortality in infections by *S. aureus*, but its meaning in the prognosis of *S. epidermidis* bacteremia is unclear [13].

Infections by *S. aureus* and CoNS behave differently, and evidence regarding *S. aureus* is not always applicable to CoNS. However, research on bloodstream and endovascular infections is often focused on *S. aureus*, which leads to available research on CoNB being more scarce. This leaves physicians to sometimes extrapolate knowledge from *S. aureus* bacteremia where there is a void of evidence.

Vancomycin is the recommended first-line therapy for methicillin-resistant *S. aureus* (MRSA) and methicillin-resistant CoNS (MR-CoNS) BSI [14,15]. In prosthetic-valve infective endocarditis (IE) by methicillin-resistant staphylococci, a combination of an aminoglycoside and rifampicin has traditionally been used [16]. Daptomycin has shown non-inferiority compared to vancomycin in complicated BSI and IE by MRSA and MR-CoNS [17-19]. In IE, when vancomycin cannot be used or is not tolerated by the patient, high-dose daptomycin (≥10 mg/kg) is the second-line choice of therapy, in association with a second agent chosen according to antimicrobial susceptibility testing [14,16]. There are no clear guidelines on the best next-line options when treatment with vancomycin or daptomycin fails, in complicated BSI or IE by methicillin-resistant staphylococci. Cotrimoxazole has good activity on MR staphylococci but should preferably be used as a step-down option in bacteremia, as it was associated with numerically higher mortality (albeit non-significant) when compared to vancomycin in the treatment of MRSA severe infections [20]. Linezolid has shown promising results as salvage therapy for persistent MRSA bacteremia, but its prolonged use is limited by toxicity [21,22].

We presented a small case series of MRSE-complicated endovascular infections treated with the DAP + CFT combination therapy, mostly as salvage therapy after first-line treatment failure, a situation for which there are no clear guidelines in the literature. The association of DAP + CFT was based on growing evidence that this combination therapy might offer some benefits in MRSA infection either as salvage therapy after first-line treatment failure or in the subgroup of patients with complicated primary endovascular infections.

A retrospective case series of 10 patients with persistent MRSA BSI, in which combination therapy with VAN or DAP plus CFT was used as salvage therapy, showed microbiologic cure in all patients and, in those with persistently positive blood cultures, sterilization of blood cultures in a median time of three days after switching to combination therapy. None of those patients had complete source control at the time that blood culture sterility was achieved [1]. A retrospective matched-cohort study of 171 patients comparing the outcomes of 113 patients treated with SOC monotherapy and 58 patients treated with the DAP + CFT combination therapy at any time during treatment showed no statistical difference in 30-day mortality between the two groups. The only exception was the subgroup of patients with complicated primary endovascular infections who were treated with DAP + CFT in the first 72 hours, in which an 80% decrease in mortality was observed, suggesting that the benefit of combination therapy might be higher in complicated endovascular infections [3]. A 2021 single-center retrospective cohort study that included 60 patients with MRSA-BSI with delayed blood culture clearance at 48 hours after initiation of appropriate therapy compared 30 patients receiving DAP + CPT for ≥48 hours with 30 patients receiving SOC treatment (defined as VAN or DAP ± gentamicin and/or rifampicin) [4]. Clinical failure, which was defined as a composite of MRSA-related mortality or recurrence of infection at 60 days, was not statistically different in both groups, although it was numerically lower in the DAP + CFT combination therapy group (20% in the DAP + CFT group vs. 43% in the SOC group), mainly due to a reduction in the number of recurrences in the combination group, whereas MRSA-related mortality at 60 days was similar in both groups.

Based on the available evidence for the DAP + CFT combination therapy in MRSA bacteremia, when faced with MRSE difficult-to-treat endovascular infections, considering the similarities between staphylococcal infections, it is reasonable to extrapolate these data and apply the same strategy to these patients. Even though *S. epidermidis* usually behaves in a less aggressive manner than *S. aureus*, which could lead to potential therapeutic benefits in SAB not being reproducible in CoNS, the severity of these MRSE infections might overcome that difference.

The mortality reflected in this case series may largely be due to baseline patient factors, as shown by the fact that all three patients who died had a CCI of 6. One of these patients died of progression of MRSE endocarditis despite DAP + CFT; the other two died of superimposition of different infections. Of the four patients who survived, none showed relapse or death at 12 months after treatment completion. 

Source control is of paramount importance, although not always possible to achieve in an ideal timing (or even not possible at all). In this case series, endocarditis patients were fragile and with high surgical risk; therefore, surgical valve replacement was often delayed in the hope that antimicrobial therapy alone would resolve the infection. In some patients, the possibility of surgical source control was altogether excluded from the start. This led to the escalation of antimicrobial therapy in cases where surgery might have sufficed. There were also cases in which combination therapy was used as a bridge of salvage therapy until source control could be achieved, therefore making it doubtful if a positive outcome was due to the combination therapy itself or rather to finally achieving source control.

The only case of severe adverse reactions was seen in the patient who developed severe neutropenia, which resolved promptly with drug interruption. In the other two cases, therapy was discontinued merely out of precaution. Of note, these patients had all been under DAP + CFT for at least four weeks, longer than in previous examples available in the literature in which this combination therapy was used.

This study has important limitations, starting with the fact that it is a retrospective study with very few patients, which reflects how unfrequent the use of the DAP + DCT combination therapy is for MRSE infections. Moreover, of the four patients who survived, two were found to already have negative blood cultures prior to the switch and another was submitted to cardiac valve replacement prior to the switch, which might mean that the source control was the true modifying factor, rather than the optimized antimicrobial therapy. Therefore, the results shown in this series do not allow to recognize the role that combination therapy with DAP + CFT truly played in the patients who survived.

## Conclusions

This small case series is meant to share our center’s experience with using combination therapy with DAP + CFT in complicated endovascular MRSE infections. It has so far been used infrequently, mostly as salvage therapy in patients with persistent MRSE bacteremia or progression of endocarditis under first-line antimicrobial therapy. 

There are no clear guidelines on the best approach after the failure of first-line treatment. Thus far, linezolid has shown promising results, but its use is limited by toxicity, especially in long treatments required for endocarditis. Combination therapy with daptomycin and ceftaroline has shown positive results as salvage treatment in MRSA infections, hinting that it might have a role in complicated MRSE infections. From our limited experience with this option, considering the poor baseline functional status of most included patients, the severity of the infection, and the fact that this combination was used as salvage therapy in the majority of patients, as well as the low rate of serious adverse events and the apparent full resolution of infection in the patients who survived, we consider DAP + CFT combination therapy to be a valid approach in these difficult-to-treat infections.

## References

[1] Hornak JP, Anjum S, Reynoso D (2019). Adjunctive ceftaroline in combination with daptomycin or vancomycin for complicated methicillin-resistant Staphylococcus aureus bacteremia after monotherapy failure. Ther Adv Infect Dis.

[2] Geriak M, Haddad F, Rizvi K (2019). Clinical data on daptomycin plus ceftaroline versus standard of care monotherapy in the treatment of methicillin-resistant Staphylococcus aureus bacteremia. Antimicrob Agents Chemother.

[3] McCreary EK, Kullar R, Geriak M (2020). Multicenter cohort of patients with methicillin-resistant Staphylococcus aureus bacteremia receiving daptomycin plus ceftaroline compared with other MRSA treatments. Open Forum Infect Dis.

[4] Johnson TM, Molina KC, Miller MA, Kiser TH, Huang M, Mueller SW (2021). Combination ceftaroline and daptomycin salvage therapy for complicated methicillin-resistant Staphylococcus aureus bacteraemia compared with standard of care. Int J Antimicrob Agents.

[5] Kleinschmidt S, Huygens F, Faoagali J, Rathnayake IU, Hafner LM (2015). Staphylococcus epidermidis as a cause of bacteremia. Future Microbiol.

[6] Flemming HC, Wingender J, Szewzyk U, Steinberg P, Rice SA, Kjelleberg S (2016). Biofilms: an emergent form of bacterial life. Nat Rev Microbiol.

[7] Diekema DJ, Pfaller MA, Schmitz FJ, Smayevsky J, Bell J, Jones RN, Beach M (2001). Survey of infections due to Staphylococcus species: frequency of occurrence and antimicrobial susceptibility of isolates collected in the United States, Canada, Latin America, Europe, and the Western Pacific region for the SENTRY Antimicrobial Surveillance Program, 1997-1999. Clin Infect Dis.

[8] Archer GL, Climo MW (1994). Antimicrobial susceptibility of coagulase-negative staphylococci. Antimicrob Agents Chemother.

[9] Koksal F, Yasar H, Samasti M (2009). Antibiotic resistance patterns of coagulase-negative staphylococcus strains isolated from blood cultures of septicemic patients in Turkey. Microbiol Res.

[10] Pedroso SH, Sandes SH, Filho RA (2018). Coagulase-negative Staphylococci isolated from human bloodstream infections showed multidrug resistance profile. Microb Drug Resist.

[11] Latif M, Usman J, Gilani M (2015). Coagulase negative staphylococci - a fast emerging threat. J Pak Med Assoc.

[12] Ryffel C, Tesch W, Birch-Machin I (1990). Sequence comparison of mecA genes isolated from methicillin-resistant Staphylococcus aureus and Staphylococcus epidermidis. Gene.

[13] N'Guyen Y, Baumard S, Vernet-Garnier V, Batalla AS, de Champs C, Jaussaud R, Strady C (2012). Coagulase-negative Staphylococcus bacteraemia accounts for one third of Staphylococcus bacteraemia in a French university hospital. Scand J Infect Dis.

[14] Brown NM, Goodman AL, Horner C, Jenkins A, Brown EM (2021). Treatment of methicillin-resistant Staphylococcus aureus (MRSA): updated guidelines from the UK. JAC Antimicrob Resist.

[15] Liu C, Bayer A, Cosgrove SE (2011). Clinical practice guidelines by the infectious diseases society of america for the treatment of methicillin-resistant Staphylococcus aureus infections in adults and children. Clin Infect Dis.

[16] Habib G, Lancellotti P, Antunes MJ (2015). 2015 ESC Guidelines for the management of infective endocarditis: The Task Force for the Management of Infective Endocarditis of the European Society of Cardiology (ESC). Endorsed by: European Association for Cardio-Thoracic Surgery (EACTS), the European Association of Nuclear Medicine (EANM). Eur Heart J.

[17] Fowler VG Jr, Boucher HW, Corey GR (2006). Daptomycin versus standard therapy for bacteremia and endocarditis caused by Staphylococcus aureus. N Engl J Med.

[18] Levine DP, Lamp KC (2007). Daptomycin in the treatment of patients with infective endocarditis: experience from a registry. Am J Med.

[19] Carugati M, Bayer AS, Miró JM (2013). High-dose daptomycin therapy for left-sided infective endocarditis: a prospective study from the international collaboration on endocarditis. Antimicrob Agents Chemother.

[20] Paul M, Bishara J, Yahav D (2015). Trimethoprim-sulfamethoxazole versus vancomycin for severe infections caused by meticillin resistant Staphylococcus aureus: randomised controlled trial. BMJ.

[21] Park HJ, Kim SH, Kim MJ (2012). Efficacy of linezolid-based salvage therapy compared with glycopeptide-based therapy in patients with persistent methicillin-resistant Staphylococcus aureus bacteremia. J Infect.

[22] Jang HC, Kim SH, Kim KH (2009). Salvage treatment for persistent methicillin-resistant Staphylococcus aureus bacteremia: efficacy of linezolid with or without carbapenem. Clin Infect Dis.

